# Developing Smokeless Tobacco Prevention Messaging for At-Risk Youth: Early Lessons from “The Real Cost” Smokeless Campaign

**DOI:** 10.1089/heq.2018.0029

**Published:** 2018-08-01

**Authors:** Matthew W. Walker, Sarah A. Evans, Cameron Wimpy, Amanda T. Berger, Alexandria A. Smith

**Affiliations:** ^1^ Center for Tobacco Products, U.S. Food and Drug Administration (FDA) Silver Spring, Maryland.; ^2^ Squared Research, Fairfax, Virginia.; ^3^ Department of Political Science, Massachusetts Institute of Technology, Cambridge, Massachusetts.; ^4^ Battelle, Health Unit, Washington, DC.

**Keywords:** adolescent, rural, smokeless, tobacco

## Abstract

**Introduction:** Smokeless tobacco (SLT) use continues to be a significant public health challenge in the United States, particularly among young males in rural areas, where use remains disproportionately high. In support of the U.S. Food and Drug Administration's first nationwide SLT public education campaign, formative research was conducted to inform campaign strategy development and test creative concepts.

**Methods:** Qualitative research methods were used to inform the strategic direction of the campaign, identify salient message themes, and refine creative concepts. Focus groups were conducted with 252 rural male youth ages 12–17 in seven states. Groups were organized by SLT status (i.e., at-risk for initiating vs. experimenting with SLT) and age group.

**Results:** SLT use is culturally ingrained in rural communities, and rural youth are commonly exposed to SLT through close relationships. Among this group, “dipping” (SLT use) has strong cultural significance and is perceived as safe. Members of the target audience are receptive to straightforward facts delivered by authentic messengers about the potentially harmful consequences of SLT use, specifically those that leverage the progression of short-term consequences (e.g., white patches) to long-term health effects.

**Conclusions:** This study addresses SLT literature gaps related to youth knowledge, attitudes, and beliefs by summarizing audience learnings from formative research that was used to develop the first national SLT public education campaign.

## Introduction

Use of smokeless tobacco (SLT), including chewing tobacco, snuff, dip, snus, and/or dissolvable tobacco, remains a public health problem in the United States. Researchers have identified more than 30 carcinogens in various SLT products, and every year more than 2,300 people in the United States are diagnosed with oral, esophageal, and pancreatic cancer because of SLT use.^1^ While the national current established use rate for all adults is below 3%, significant differences in patterns of use exist and rates among certain subpopulations can be much higher than the national average.^2^ Among adults, current SLT use is most common among men (5.7%), younger adult males ages 18–34 years (4.0%), and non-Hispanic whites (3.9%).^3^ In addition, use is particularly elevated in rural areas where SLT use rates among nonurban residents (7.9%) exceeds that of urban areas (2.3%).^3^ Initiation of SLT use typically starts at a young age with nearly 8 out of 10 (77%) of adult daily SLT users first having tried the product by age 18.^4^ Nationwide, 5.8% of all high school students and 8.3% of male high school students used SLT on at least 1 day during the past thirty days.^5^ This nationwide prevalence corresponds to ∼860,000 high school students who currently use SLT products, and each day in the United States, more than 750 male youth younger than 18 use SLT for the first time.^6^

There are several factors that increase the likelihood that teens will begin experimenting with SLT use. Initiation among male youth has been shown to be influenced by their fathers, grandfathers, male cousins, and brothers, and tobacco companies spend billions of dollars marketing tobacco products to these vulnerable populations each year.^7^ In 2015, the tobacco industry spent $684.9 million on SLT advertising alone.^8^ In recognition of the problem, the 2014 Surgeon General's Report stated that SLT use continues to be a significant public health challenge, underscoring the need for a concentrated effort to inform the public about the dangers of SLT use.^9^

### Background: “The Real Cost” smokeless tobacco education campaign

Evidence from controlled field experiments and population studies demonstrates that mass media campaigns designed to discourage tobacco use can change attitudes about tobacco use and reduce smoking prevalence.^10–13^ Furthermore, when campaigns leverage messages that are tailored to the target audience, compared with more general messaging, they can increase the likelihood of change in health behaviors.^14,15^

In June 2009, the Family Smoking Prevention and Tobacco Control Act was enacted, granting the U.S. Food and Drug Administration (FDA) the authority to regulate tobacco products and educate the public about the risks associated with tobacco use.^16^ As part of this authority, FDA launched “The Real Cost” campaign in February 2014 to educate young people ages 12–17 about the dangers of cigarettes. In April 2016, FDA expanded “The Real Cost” to include new advertising targeting rural male youth at risk of SLT use.

### Defining the target audience

Research indicates that rural youth are disproportionately affected by SLT use. According to an analysis of the National Survey on Drug Use and Health, SLT use is more than twice as likely in rural areas compared with metropolitan areas in the United States, and living in a rural area is considered a key risk factor for SLT use because of cultural, educational, and environmental factors.^4,17^ Rural male teenagers see SLT being used by role models such as fathers, grandfathers, older brothers, and community leaders, and as a result, SLT use has become culturally ingrained in rural communities and is seen as a normalized rite of passage.^18^ However, some rural youth are impacted more significantly than others–with white, male youth being more likely to use SLT than other youth. According to the most recent data from FDA's Population Assessment of Tobacco and Health study, SLT use was found to be most common among nonurban, white (non-Hispanic) males.^6^ For these reasons, initial education efforts for “The Real Cost” Smokeless campaign were focused on developing evidence-based messages that would resonate with rural, white (non-Hispanic) male youth.

### The current study

This study focuses on broad learnings related to rural youth SLT use that were uncovered through two formative studies conducted to support the strategic and creative development of health messaging for “The Real Cost” Smokeless campaign. The findings described are a result of two distinct phases of campaign formative research (strategic concept testing and creative concept testing).

## Methods

### Design and procedures

To inform strategic and creative direction for “The Real Cost” Smokeless campaign, the FDA conducted focus group research with members of the campaign target audience. Two phases of research were conducted, during which 41 focus groups were held with 252 participants. Recruitment took place in states with high SLT prevalence and high proportions of the target audience. The research team comprised FDA and contractor staff, including members of the advertising agency contracted to help develop the campaign (The Sensis Agency) and a research organization that assisted with on-the-ground research, analysis, and reporting (Fors Marsh Group). Both study protocols were reviewed and approved by FDA's Institutional Review Board.

### Sample selection

Reflecting the campaign target audience, participants were rural, white (non-Hispanic), male youth, ages 12–17, who were at risk for initiating or already experimenting with SLT. “At-risk” was defined based on established susceptibility items^19^ adapted for SLT use. Youth who reported that they had never tried SLT were asked three items to assess future intentions to use: (1) Do you think you will use chewing tobacco, snuff, or dip soon? (2) Do you think you will use chewing tobacco, snuff, or dip in the next year? (3) If one of your best friends were to offer you chewing tobacco, snuff, or dip, would you use it? Response options included “definitely yes,” “probably yes,” “probably not,” or “definitely not.” Youth who gave a response other than “definitely not” to any of the three items were defined as “at-risk.” Participants who self-reported using SLT less than 100 times in their lifetime along with not reporting current daily use (more than five times per week in the previous 30 days) were classified as “experimenters” and eligible for inclusion. The two qualitative studies referenced below were conducted with youth who met the criteria for being either “at-risk” or an “experimenter.” Youth who had never tried SLT and were not susceptible were excluded, as were youth who were established or current, daily users. Focus groups consisted of roughly six participants per group and were separated by age (12–14 and 15–17) and SLT use status (at-risk and experimenter).

### Strategic concepts focus groups

Based on insights from literature reviews and in consultation with subject matter experts, six strategic concepts were developed to educate rural youth about the health consequences of SLT use. To obtain target audience feedback on the strategic concepts, 15 focus groups were conducted in the spring of 2014 with 106 members of the target audience in Arkansas, Iowa, Kansas, and Oklahoma (see Table 1 for sample characteristics). Focus groups were conducted in market research facilities, and youth were recruited by phone via existing facility databases of individuals interested in research participation. The focus groups lasted 90 min and were audiorecorded, transcribed, and qualitatively analyzed. Participants were asked to provide feedback on each concept, including comprehension of the main message; believability; relevance; powerful words and phrases; and what they would change to make it better.

**Table 1.** **Sample Characteristics**

			Number of participants		
Round location	Age range	Number of focus groups	At-risk	Experimenter	Total
*Strategic concepts*					
Arkansas	12–14	1	8	0	8
	15–17	3	8	16	24
Iowa	12–14	1	8	0	8
	15–17	3	8	9	17
Kansas	12–14	2	16	0	16
	15–17	2	5	7	12
Oklahoma	12–14	1	8	0	8
	15–17	2	8	5	13
**Total**	12–14	5	40	0	40
	15–17	10	29	37	66
	**Total**	**15**	**69**	**37**	**106**
*Creative concepts*					
Arkansas	12–14	3	13	6	19
	15–17	2	0	11	11
Iowa	12–14	2	10	0	10
	15–17	4	11	11	22
Montana	12–14	1	7	0	7
	15–17	4	12	10	22
Virginia	12–14	1	6	0	6
	15–17	4	9	11	20
Wisconsin	15–17	5	12	17	29
**Total**	12–14	7	36	6	42
	15–17	19	44	60	104
	**Total**	**26**	**80**	**66**	**146**

### Creative concepts focus groups

Based on insights from the strategic concepts research, seven creative concepts were developed in the form of “animatics” (animated story boards with voice-overs). In the spring of 2015, the creative concepts were tested in 26 focus groups with 146 participants in Arkansas, Iowa, Montana, Virginia, and Wisconsin (see Table 1 for sample characteristics). Youth were recruited in schools, and 60- to 90-min focus groups were conducted either during or after school. The focus groups explored initial reactions to concepts, main messages, persuasiveness, relatability of the main character, and reactions to facts about health consequences. The focus groups were audio-recorded, transcribed, and qualitatively analyzed.

### Data analysis

The research team used a systematic qualitative analysis process throughout both phases of research, which included qualitatively analyzing participant reactions to each concept; comprehension of the main message; believability; relevance; powerful words and phrases; and general feedback on what participants would change to make it better.

For the strategic concepts phase, transcripts were systematically analyzed using a grounded theory approach. For the creative concepts phase, focus group transcripts were first organized into broad categories that related to specific research questions and probes on the moderator guides. In both studies, emergent themes were categorized to allow for a rich description of findings.

## Results

This research resulted in campaign-specific implications for the continued development and refinement of FDA's advertisements. Although the strategic concepts study and the creative concepts study were conducted during two different stages of the campaign development process as two separate research studies, the emergent themes from the two phases were largely consistent. Thus, results of the two studies are combined unless otherwise noted. These results are detailed below and summarized in Table 2.

**Table 2.** **Summary of Key Findings**

Finding	Manifestation in rural, SLT context	In their own words…
Message authenticity is critical.	• Strong cultural influences with regard to SLT use. • Nuances in language, settings, imagery, and characters have significant power to distract from or enhance the underlying message. • Care must be taken to not portray rural boys' lifestyle or culture in stereotypical ways.	“… he looks like a city person.” (*12–14 At-Risk)* “… not realistic to be spitting in a cup.” *(15–17 Experimenter)* “I like that message but the execution could be better than the small-town thing.” *(15–17 Experimenter)*
Relative risk perceptions influence attitudes and decision-making.	• Rural youth hold strong opinions regarding the relative harm of SLT use compared with cigarettes. • Exaggerated health consequences were a reason to dismiss SLT prevention messaging.	“[SLT] is a little better because cigarettes can actually destroy your lungs.” *(12–14 At-Risk)* “You are talking about dip like it's crack.” *(15–17 Experimenter)*
Long-term health consequences can be too abstract to impact behavior, particularly among youth.	• Certain short-term, visible effects are nearly universally accepted as part of SLT use (e.g., sore gums, white patches). • More serious health effects were generally dismissed as highly unlikely or too long term. • Familiar effects provided an avenue to talk about more serious long-term consequences as a progression from short-term effects.	“…hearing small, white patches before I thought maybe that wasn't a big deal. Like, oh, I can have a little patch of white in my mouth. It's not going to make a big difference but, like, that's the first step to cancer. It'd definitely make you think twice.” *(15–17 Experimenter)* “I really liked it because it explains like what it does to you and it explains like where it can start.” *(12–14 At-Risk)*
Youth gravitate toward facts and value their ability to make their own informed decisions.	• Youth were receptive to messages containing concrete facts • Participants were quick to pick up on qualifying language, such as “may cause.”	“I appreciate how they put on the facts…because the facts are what make it stick out to me, and…also, the statements make it more memorable.” *(12–14 At-risk)* “I think that it uses the word like ‘might’ too much. Like your gums and lips might get sores on them… It makes it seem more, like, less threatening…” *(15–17 Experimenter)*

SLT, smokeless tobacco.

### Authenticity

Study participants wanted to see a world reflected that looks like their own and demanded authenticity in how they and their community are portrayed. Participants were quick to point out images that they perceived to be unrealistic and to spot differences in scenarios, settings, and character attributes that did not fit into their world (e.g., being “too city” or “too preppy”). It was common to hear comments such as “he looks like a city person” or “…not realistic that he would walk away from the [football] huddle like that.” Participants also noted when settings and characters were portrayed in a way that was overly stereotypical or “othering” (defined as perceiving someone to be fundamentally different in a way that is negative and dehumanizing) of a rural boy's lifestyle or culture. For example, the term “small town” was not well received by the target audience and was instead rejected as a dated descriptor of the rural community. Also, the use of regional accents was ridiculed as trying to “sound rural.” In one of the early concepts, using a Southern accent limited geographic appeal instead of portraying authenticity, and worse, seemed to distract from the message of the advertisement.

Just as participants would point out a seeming lack of authenticity, they also would give credit to situations or depictions of characters they judged as accurate and were quick to pick up subtle cues such as a photo depicting bass fishing or a baseball trophy in the background.

### Cultural significance of SLT

Participants reported strong social identities stemming from close ties to family, friends, and community, and in rural communities SLT can hold a strong cultural significance as a time-honored tradition. Participants revealed key sociocultural beliefs, indicating they viewed SLT use as a way to belong and a rite of passage, often modeled by adult male influencers (e.g., fathers, brothers, coaches). SLT use was considered a masculine act often introduced during male-centered activities, such as sport events, and outdoor leisure activities, such as fishing and camping.

### Health consequences: relative risk

In discussing existing knowledge of SLT risks as well as perceptions of usage patterns, participants frequently made comparisons to other drugs and products, including cigarettes. Participants generally assumed SLT is safer than cigarettes and other drugs, a perception that often served as a means to dismiss the risks altogether, such that perceptions of “safer than…” in practice were often equated as “safe” in general. The perception of reduced relative risk essentially resulted in a general reluctance to acknowledge the potentially serious health consequences of SLT use in absolute terms. Concrete health consequence facts specific to SLT showed the potential to debunk the perception that “safer than cigarettes” means “safe.” These findings indicate that while there is room to move baseline perceptions of absolute risk, portraying health consequence messages too viscerally may be perceived as exaggerated, and thereby dismissed as unrealistic.

### Health consequences: seeds of doubt

Messages portraying health consequences of SLT use resonated when framed in a progression from familiar, short-term effects to more serious long-term consequences. Long-term consequences, when discussed in isolation, were frequently cited as too abstract, too unlikely, or too far down the road for attention in the near term. Conversely, participants indicated they were highly motivated by the short-term health consequences (e.g., cavities and lesions). Participants were acutely aware of the potential for SLT users to develop mouth lesions in the form of sores or white patches on their gums, some having experienced these effects themselves. Messages connecting immediate, familiar, and outwardly visible effects with more serious long-term health consequences resonated with participants, indicating that helping youth to make this type of connection can be a critical tool for youth-focused SLT education.

### Health consequences: new and reframed information

Participants knew very little about the harmful constituents that are in SLT or about the harmful consequences of use. Participants were receptive to and frequently surprised by facts and wanted clear information on the health consequences of SLT use; however, important nuances emerged regarding how health consequences were communicated.

Participants noted the use of words such as “may” softens the language substantially and makes the statements easier to dismiss. While participants generally sought scientific information, they consistently identified medical terms, such as “lesions,” “carcinogen,” and “periodontitis” as being confusing, underscoring a particular challenge in communicating scientific information to youth in terms they understand. Participants suggested using more common language, such as “sores,” “cancer,” and “gum disease,” rather than overly technical or medical terms. In addition, although participants had heard of nicotine and knew it was linked to addiction, they did not understand exactly what nicotine does. The broad takeaway was that accuracy to details mattered and authenticity was an important factor for the performance of any given concept.

## Limitations

The limitations of this study are related to its qualitative nature—these studies were intended to provide insights and direction, not absolute measures nor a quantitative assessment projectable to a larger population. The comments made in this article are based on information gathered from a relatively small sample of respondents who were selected to participate because they met specific recruiting criteria reflective of FDA's rural youth target audience. The findings presented in this report are intended to offer a more thorough understanding of the specific issues explored in the research and, without quantitative support, cannot confidently be projected to a larger population of similar respondents. Although the results are not statistically generalizable, a diverse sample of participants was recruited with regard to SLT use status, age, and geography. Furthermore, the total sample size (*N*=252 across two studies) was adequate to reach a point of data saturation sufficient for drawing reliable thematic conclusions.

## Discussion

There is a clear need in rural markets where SLT use is a cultural norm for messaging focused on educating at-risk youth. The findings from this research have served as the foundation for FDA's “The Real Cost” Smokeless campaign and might be leveraged in other rural youth-focused SLT conversations, localized messages, and education.

Rural youth are well versed in the dangers of cigarette use, and participants in this study were quick to dismiss consequences of SLT use as “lesser than” cigarettes and made fewer connections between SLT use and addiction and serious health consequences. Similar to the evolution of smoking prevention messages, findings from this research indicate that it may be necessary in the design of youth-focused SLT prevention messages to first establish the foundation of health consequences and addiction.

Public health officials and communicators intending to design messages and education to address this need face literature gaps related to baseline knowledge, attitudes, and behaviors in the context of youth SLT use, particularly compared with the use of cigarettes. The current findings point to promising messaging areas and strategies as well as potential obstacles for addressing SLT use with rural youth when developing educational materials, tailored communication efforts, and interpersonal conversations.

## Conclusions

The present study addresses literature gaps related to rural youth knowledge, attitudes, and behaviors related to SLT. Evidence from these studies points to both unique challenges related to developing youth-focused SLT messaging and key techniques for engaging with youth about the dangers of SLT use. Less readily available in the literature is a deeper, more actionable understanding of what authenticity means and looks like for this audience as related to SLT use. This study fills some of those critical gaps by highlighting several key crosscutting learnings as well as manifestations in the context of rural SLT use.

Rural youth have strong, existing, positive beliefs about SLT that should be countered in messaging; however, care must be taken not to disregard the cultural significance of SLT in rural communities when communicating health information. Health messaging designed to educate youth about the dangers of SLT use that fails to acknowledge the sociocultural significance of SLT among this target audience may miss the mark, or at worst, be outright rejected.

Three key themes emerged with regard to how public health officials and communicators might best address the health consequences of SLT use with rural youth: (1) youth in the research often translated “safer than cigarettes” into “safe,” and frequently cited comparative harm as a reason to dismiss SLT prevention messaging; (2) there were short-term and outwardly visible effects that were nearly universally accepted as part of SLT use (e.g., developing white patches in the mouth)—these familiar effects provided a way in to talk about potential longer term effects that were more easily dismissed when presented alone; and (3) rural youth participants were receptive to and compelled by SLT facts related to health consequences when presented in common language.

## Abbreviations Used

FDA: Food and Drug Administration
SLT: smokeless tobacco
